# Designing an experimental method for assessing biocompatibility of circuit coatings using biomarkers for platelet activation during cardiopulmonary bypass

**DOI:** 10.1051/ject/2024003

**Published:** 2024-06-18

**Authors:** Meghal Sancheti, Mitchell Rentschler, Charlotte Bolch, Weidang Li, Katelyn Necco, Thomas Rath, Mitra Esfandiarei, Nathaniel Darban

**Affiliations:** 1 Arizona College of Osteopathic Medicine, Midwestern University 19555 N. 59th Avenue Glendale AZ 85308 USA; 2 Office of Research & Sponsored Program, Midwestern University 19555 N. 59th Avenue Glendale AZ 85308 USA; 3 College of Veterinary Medicine, Midwestern University 19555 N. 59th Avenue Glendale AZ 85308 USA; 4 College of Graduate Studies, Midwestern University 19555 N. 59th Avenue Glendale AZ 85308 USA; 5 College of Health Sciences, Midwestern University 19555 N. 59th Avenue Glendale AZ 85308 USA; 6 College of Medicine Phoenix, University of Arizona 19555 N. 59th Avenue Glendale AZ 85308 USA; 7 Faculty of Medicine, University of British Columbia 2176 Health Sciences Mall Vancouver BC V6T 2A1 Canada

**Keywords:** Cardiopulmonary bypass, Platelets, Biomarkers, Platelet activation, ELISA, Circuit coating

## Abstract

*Introduction*: Cardiopulmonary bypass is an essential component of cardiothoracic surgeries. However, significant complications such as systemic inflammatory response syndrome (SIRS) resulting from cardiopulmonary bypass (CPB) are a common occurrence due to contact between circulating blood and foreign surfaces that leads to platelet activation. It is suggested that different available CPB circuit coatings can potentially reduce platelet activation. However, there have been no published evidence-based reports confirming these claims. In addition, there is no well-established protocol for studying platelet activation biomarkers during CPB *in vitro* in a laboratory setting. *Methods*: CPB was simulated in the laboratory using bovine blood in two different types of coated CPB circuits: *Trillium*^®^ *Biosurface* by Medtronic, and *Xcoating*^TM^ Surface by Terumo. Fresh bovine blood samples were collected and circulated through the CPB circuit following the standard protocol used in the operation rooms. Blood samples were then collected at 5 min, 30 min, and 55 min during the circulation. Blood plasmas were separated and subjected to enzyme-linked immunosorbent assay to measure most established platelet activation markers P-selectin, Platelet Factor 4 (PF4), Glycoprotein IIb/IIIa (GPIIb/IIIa), and β-thromboglobulin (β-TG) at different time points. *Results*: The biomarker values at 30 min and 55 min were compared to the base values at 5 min for each type of CPB circuit. The results of the means from all measured biomarkers showed data measurements that indicated no significant variability within each coating. All collected data points fell within ±2 SD of the means, which was considered acceptable variations across technical replicates.  *Conclusion*: In this study, we were able to establish an *in vitro* protocol in the laboratory setting that is precise and reliable with minimum intra-variability. This established protocol will allow for future studies in which different coated CPB circuits can be compared for their effectiveness in blocking platelet activation during the CPB.

## Introduction

Cardiopulmonary bypass (CPB) provides a bloodless, motionless field for the surgeon to operate. While this has facilitated many advances in the field, it does pose significant complications, such as platelet activation and dysfunction, as well as abnormal bleeding [1]. Serious manifestations of these complications can lead to systemic inflammatory response syndrome (SIRS) and sepsis, leading to organ failure [2]. Most of these complications can be traced back to platelet activation in contact with a foreign body, such as the coating of circuits used in CPB. During the bypass, platelets bind to these artificial surfaces, leading to the release of inflammatory cytokines and activation of coagulation-fibrinolysis systems [3]. The surface markers of platelets such as platelet factor 4 (PF4), β-thromboglobulin (β-TG), glycoprotein IIb/IIIa (GPIIb/IIIa), and soluble P-selectin are released during the coagulation cascade and are considered as reliable markers for platelet activation during CPB [4].

Recent advances in bypass technologies have created biocompatible surfaces to potentially decrease platelet activation during CPB, therefore, reducing the number of complications after surgery. Heparin-coated circuits are widely used because they seem not to cause excessive platelet activation as compared to non-coated circuits [5]. Another commonly used circuit poly-2-methoxyethyl acrylate (PMEA) has a protective water layer that allows proteins to retain their conformation and reduce adherence to foreign surfaces [6].

While this has been the focus of research and advancement in cardiothoracic surgery, there is minimal scientific and data-driven evidence that establishes the biocompatibility of these circuits or their potential benefits in reducing platelet activation during CPB; hence, there has yet to be a reliable and standardized laboratory protocol that compares these biocompatible circuits *in vitro* with respect to their effects on platelet function and activation [7].

Much of our evidence of biocompatibility comes from platelet activation studies done during clinical studies with patients undergoing surgery with limited sample size and multiple independent variables [8–10]. In this study, we aimed to 1) establish a well-controlled and standardized *in vitro* protocol to measure surface markers of platelet activation at different time points during the bypass in the laboratory setting using commercially available Enzyme-Linked Immunosorbent Assay (ELISA) kits, and 2) determine the intra-coating variability across technical replicates for two types of coated circuit that are commonly used in CPB to establish the required technical replicate within each independent biological replicate.

## Materials and methods

### Bovine blood collection

Bovine blood was obtained via venipuncture from a local slaughterhouse and treated with 30,000 IU of heparin in the collection bucket to reduce clotting during sample transportation. Eight to nine (8–9) liters of bovine blood were used for one run of CPB. An activated clotting time was accepted and maintained above 480 s after heparin administration [11]. The circuit was not connected to a living system; hence no extra heparin was added during the experimental runs of CPB. Experiments with CPB were started within 1–2 h and completed within 8–9 h post-blood collection.

### CPB circuit setup

**Figure 1:**
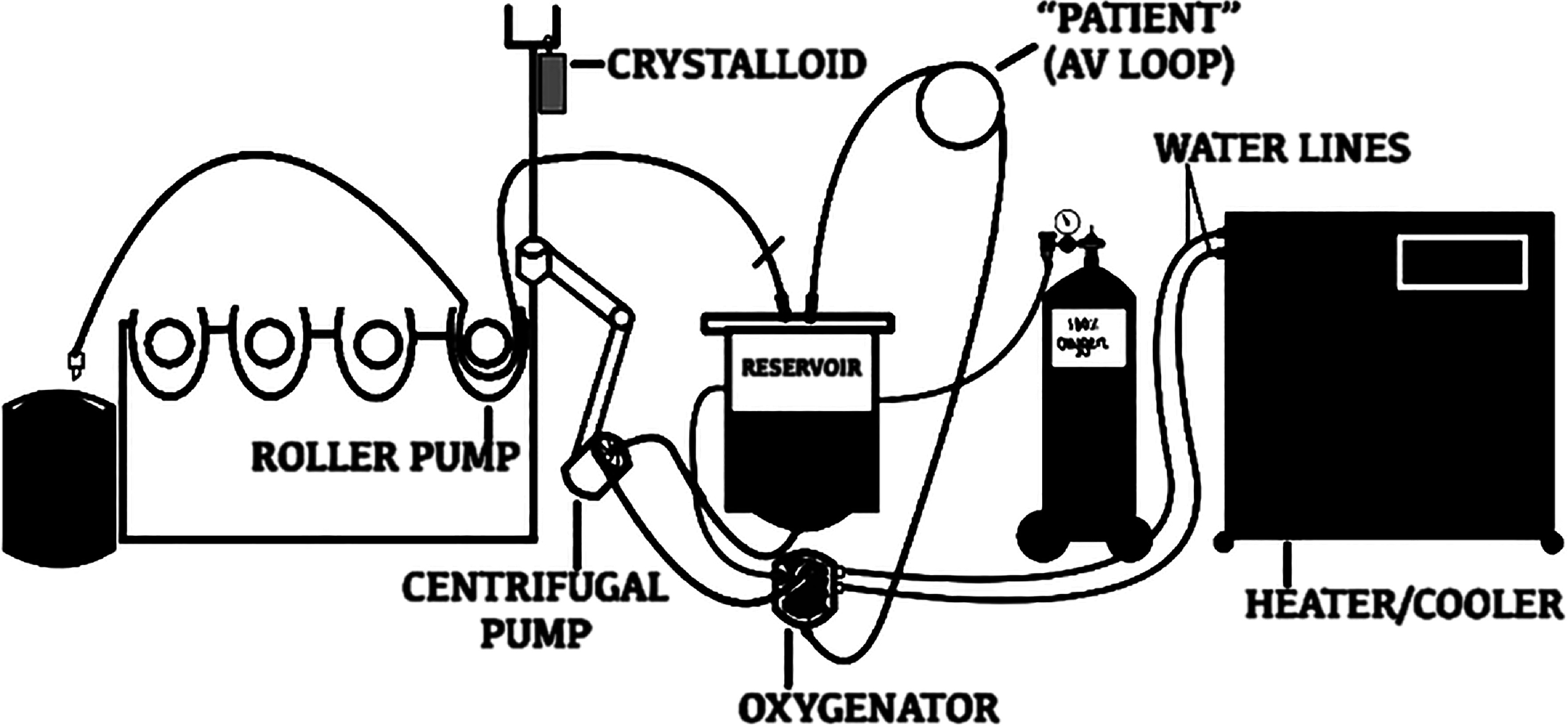
Schematic of the laboratory-based (*in vitro*) Cardiopulmonary Bypass Circuit.

One bovine blood donation was used for five technical replicates of experimental CPB runs using *Trillium*^*®*^ *Biosurface* (541T, Medtronic Inc., Minneapolis, MN) with a heparinized surface. Another bovine blood donation was used for five technical replicates of experimental CPB runs using *Xcoating*^TM^ (3CX*FX25RWC, Terumo Cardiovascular, Ann Arbor, MI), which is a PMEA-coated circuit [12]. The laboratory simulated circuit included a pump, arteriovenous (AV) loops, tubing, reservoirs, oxygenators, arterial line filters, a heat exchanger (16-02-85, SORIN Group Deutschland GmbH, Müchen, Germany), and a roller pump (Stockert S3 Roller Pump with S3 Console 10-60-00, SORIN Group Deutschland GmbH, Müchen, Germany). All parts of the circuit were replaced before each technical replicate trial (Fig. 1).

During the experimental runs, bovine blood was mixed with crystalloid (Normosol-R) to prime and de-air the CPB circuit. The amount of crystalloid used was calculated to maintain a hematocrit of 23–27%. Blood was circulated at a rate of 4 L/min and was mixed with crystalloid for the first 5 min of the run. Blood samples were collected at baseline (5 min after initiation of CPB at 37 °C), 30 min after initiation of CPB at 32 °C, and 55 min after initiation of CPB at 37 °C. The circuit run was completed in 60 min. At each desired time point, 1 mL of blood samples were collected using K2 EDTA-coated Vacutainer tubes (catalog number 367863; Becton Dickinson, San Diego, CA). Collected blood samples were centrifuged at 2500 RPM (Microfuge Lite 367121, Beckman Coulter, Inc., Palo Alto, CA) for 15 min. Separated plasma samples were aliquoted (500 μL) into small CryTubes (Thermo Scientific Nalgene System, Rochester, NY) and stored at −80 °C for ELISA assay.

### Measurements of platelet count

The whole bovine blood sample was collected in a sterile vacutainer tube containing 15% EDTA K3 solution (Covidien, Cardinal Health, 8881311446) and stored at 4 °C. Platelet counts were assessed using Siemens Advia 2120i (Siemens Medical Solutions, Inc., Malvern, PA) by a single optical cytometer after appropriate dilution of blood samples with ADVIA series RBC/PLT reagent. Platelets were counted from the signals of the common detector, and coincidence correction was made so that accurate counts were made over a wide range of cell types.

### Assessments of platelet activation biomarkers

Markers for platelet activation were measured in duplicates by ELISA assay according to instructions provided by the manufacturer (MyBioSource Inc., San Diego, CA) and at a 1:2 dilution. Collected plasma samples were used to measure four biomarkers of platelet activation: PF4 (MBS741915), β-TG (MBSO89872), P-selectin (MBS734634), and GPIIb/IIIa (MBS739298) at three different collection time points (5 min, 30 min, and 55 min). Detection ranges and sensitivity for each kit are as listed: PF4 (25–500 ng/mL and 1.0 ng/mL), β-TG (0.156–10 ng/mL and 0.05 ng/mL), P-selectin (5–100 ng/mL and 1.0 ng/mL), and GPIIb/IIIa (1–25 ng/mL and 0.1 ng/mL). The values for standards and experimental samples were then analyzed using the ELISA analysis software (BioTek ELISA Version 2.06, BioTek, Winooski, VT).

### Statistical analysis

The values obtained from the ELISA assays were averaged across the five runs within each of the two coatings for each time point at 5 min, 30 min, and 55 min. The analysis methods used were descriptive statistics to quantify potential changes in biomarker values as determined by the ELISA assays. A standard deviation (SD) from the means was calculated and the individual values of each run were compared to the mean to see if they were within ±2 SD of the mean. This method was used to determine whether any outlier values were found. Error bars were calculated as standard errors, and the results were represented as bar graphs. No statistical inference tests between two different coatings were used given the limited sample size and low statistical power. The descriptive statistics from this pilot study will be used to inform and power future study designs with adequate biological replicates.

## Results

### Quantification of platelet counts

**Figure 2:**
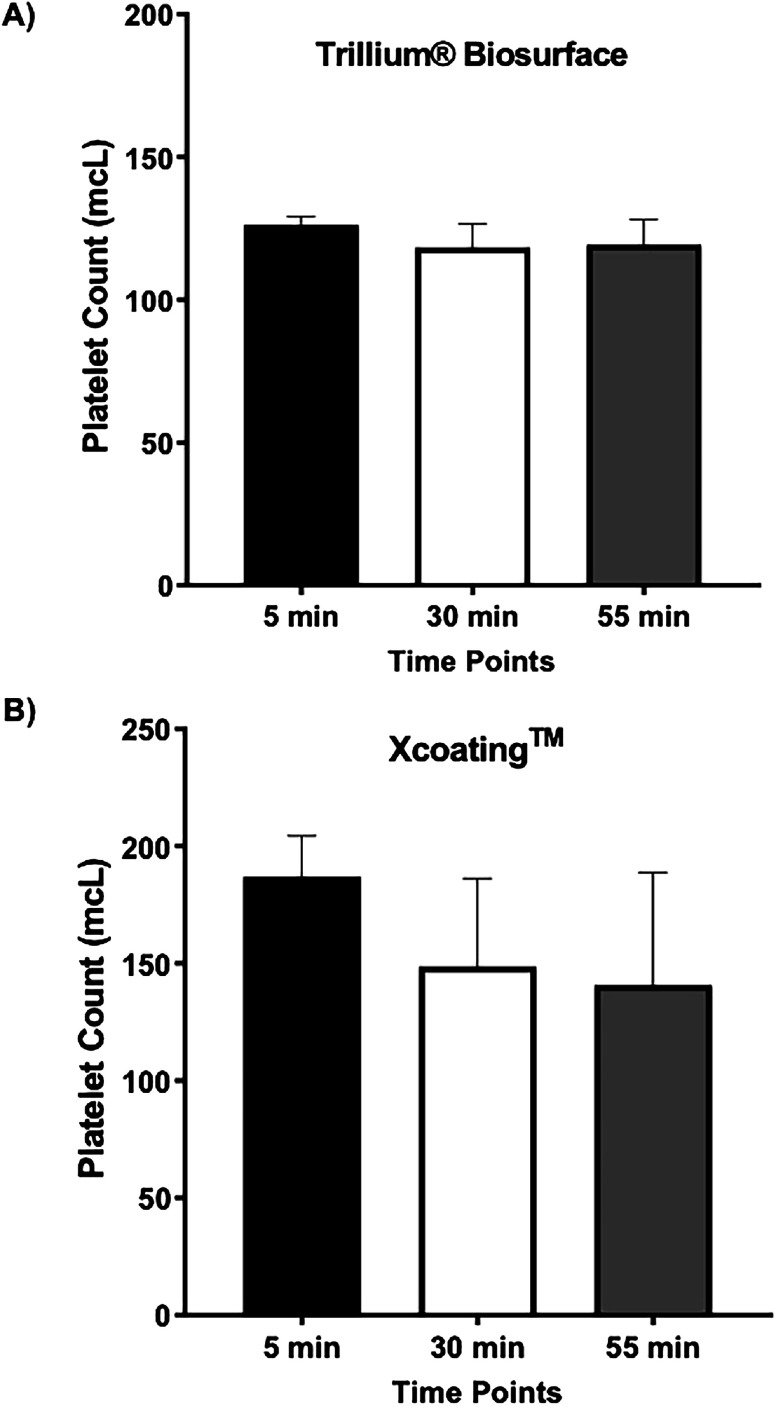
*Quantification of Platelet counts in* circulated bovine blood. (A) Bar graph presentation of platelet counts at different time points of bovine blood circulation using *Trillium*^®^ *Biosurface* circuit. Data presents the average of five technical replicates using the same blood samples at 5 min, 30 min, and 55 min with no significant changes between time points. (B) Bar graph presentation of platelet counts at different time points of bovine blood circulation using *Xcoating*^TM^ circuit. Data presents the average of five technical replicates using the same blood samples at 5 min, 30 min, and 55 min with no significant changes between time points. (Mean ± SD, *P* < 0.05, five technical replicates).

To establish a reliable *in vitro* protocol to measure platelet count post bypass using different coatings, we measured the range for inter-variation between bypass technical replicates for *Trillium*^®^ *Biosurface* and *Xcoating*^TM^ circuits, by running the same bovine blood samples five times through each type of coated circuits (Fig. 2). A new package of circuit was used for each technical replicate run (five unused circuit packages for each type of coating) and all five replicates were run during the same experimental day. Our data showed no significant increase or drop in platelet count at 5 min, 30 min, and 55 min for both coating types, with values at each time point falling within ±2 SD of the mean for *Trillium*^®^ *Biosurface* (Fig. 2A) and *Xcoating*^TM^ (Fig. 2B).

### Measurements of biomarkers of platelet activation in *Trillium*^®^ *Biosurface* circuit

**Figure 3:**
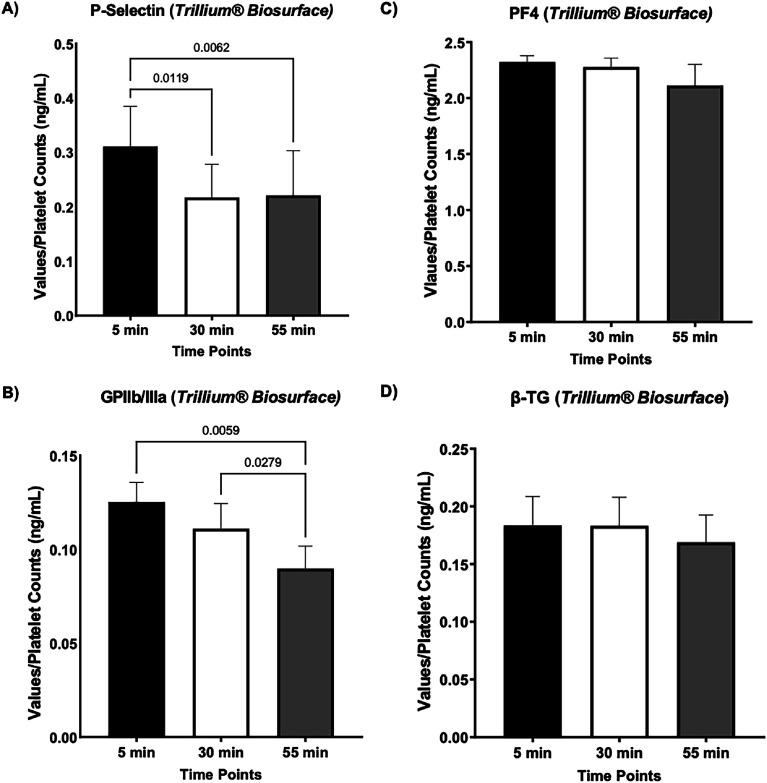
*Measurements of biomarkers of platelet activation in Trillium*^®^ *Biosurface circuit.* (A) Soluble P-selectin levels reduced at 30 min and 55 min, with values at each time point falling within ±2 SD of the mean. All measurements are presented as normalized values over the platelet count for each time points. (B) The normalized values for GPIIb/IIIa levels in circulated bovine blood show a significant decrease at 55 min, with all measured values falling within ±2 SD of the mean. (C) Normalized values for PF4 and (D) β-TG did not show any significant changes at 5 min, 30 min, and 55 min, but all values fell within ±2 SD of the mean, indicating minimal intra-variability across technical replicates. (Mean ± SD, *P* < 0.05, five technical replicates). GPIIb/IIIa: Glycoprotein IIb/IIIa; PF4: Platelet Factor 4; β-TG: β-Thromboglobulin.

**Supplementary Figure S1.**
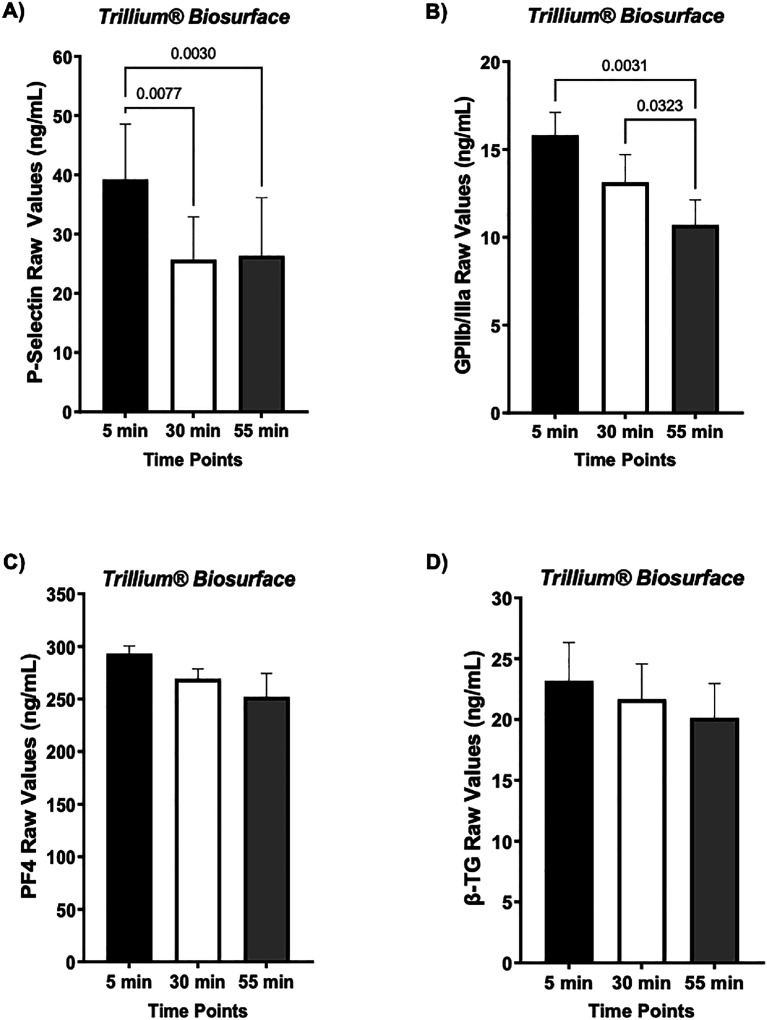
Raw values for platelet activation biomarkers using *Trillium*^®^ *Biosurface* bypass. Bar graphs presenting raw values of platelet activation markers in collected bovine blood at 5-, 30-, and 55-minutes using *Trillium*^®^ *Biosurface* bypass circuit without normalization over the platelet counts. (Mean ± SD, *P* < 0.05, five technical replicates). GPIIb/IIIa: Glycoprotein IIb/IIIa; PF4: Platelet Factor 4; β-TG: β-Thromboglobulin

ELISA assay was used to measure the levels of platelet activation markers (soluble P-selectin, PF4, GPIIb/IIIa, and β-TG) in circulated bovine blood at 5-, 30-, and 55-minutes during circulation using *Trillium*^®^ *Biosurface* coated tubes. The values for biomarkers at 5 min were arbitrarily set as the baseline measurement for each biomarker within the same circuit type. Measurements at 30 min and 55 min were then compared to measured values at 5 min to assess potential time-dependent changes in biomarkers at each collection time point. All the biomarker concentrations were normalized over the platelet counts for that time point. The results indicate that in the *Trillium*^®^ *Biosurface* circuit, the values for platelet activation biomarkers fall within ±2 SD of the mean for soluble P-selectin, GPIIb/IIIa, PF4, and β-TG (Fig. 3). The normalized values for soluble P-selectin dropped at 30 min and 55 min (Fig. 3A), with GPIIb/IIIa levels showing a significant drop only at 55 min (Fig. 3B) as compared to basal levels recorded at 5 min. On the other hand, the values for PF4 and β-TG did not show any significant changes with time (Figs. 3C and 3D). The data indicates that the luminal surface of *Trillium*^®^ *Biosurface* circuits has no effects on the secretion levels of these two biomarkers in circulated blood. We also ran statistical analyses using the recorded raw values for all four biomarkers secretion and translocation levels without normalization over the platelet counts in bypassed bovine blood at 5–, 30–, and 55–minutes in *Trillium*^®^ *Biosurface* circuit (Supplementary Fig. S1).

### Measurements of biomarkers of platelet activation in *Xcoating*^TM^ Circuit

**Figure 4:**
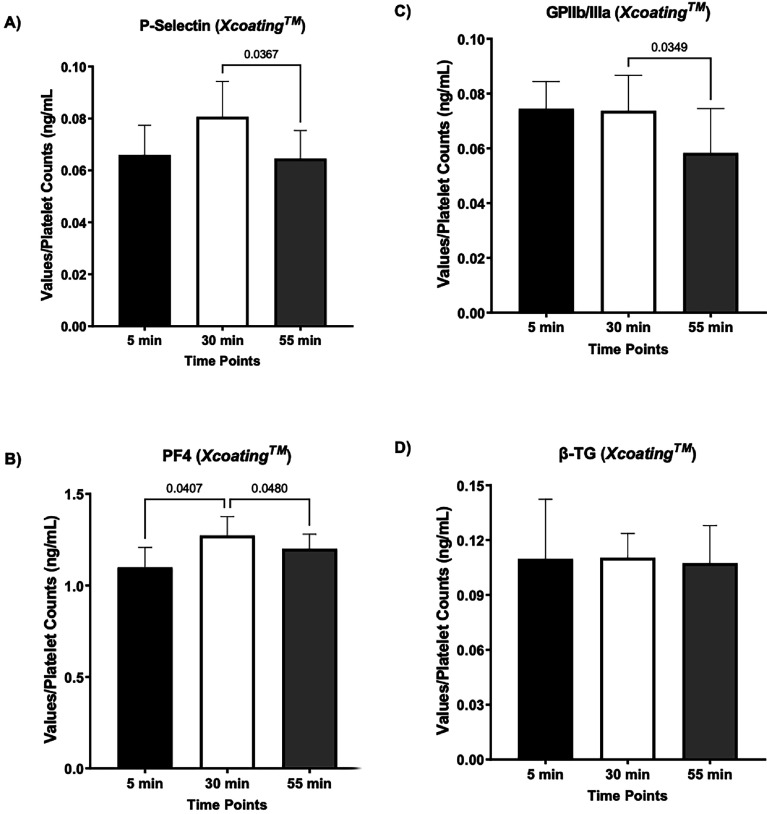
*Measurements of biomarkers of platelet activation in Xcoating*^TM^ *circuit.* (A) No significant changes were detected in soluble P-selectin levels in collected bovine blood at 5 min, 30 min, and 55 min using the *Xcoating*^TM^ circuit, with values at each time point falling within ±2 SD of the mean. All measurements are presented as normalized values over the platelet count for each time point. (B) The normalized values for PF4 did not change over time with all values at each timepoint falling within ±2 SD of the mean. (C) Normalized values for GPIIb/IIIa and (D) β-TG did not show any significant changes at 5 min, 30 min, and 55 min, but all values fell within ±2 SD of the mean, indicating minimal intra-variability across technical replicates. (Mean ± SD, *P* < 0.05, five technical replicates). GPIIb/IIIa: Glycoprotein IIb/IIIa; PF4: Platelet Factor 4; β-TG: β-Thromboglobulin.

**Supplementary Figure S2.**
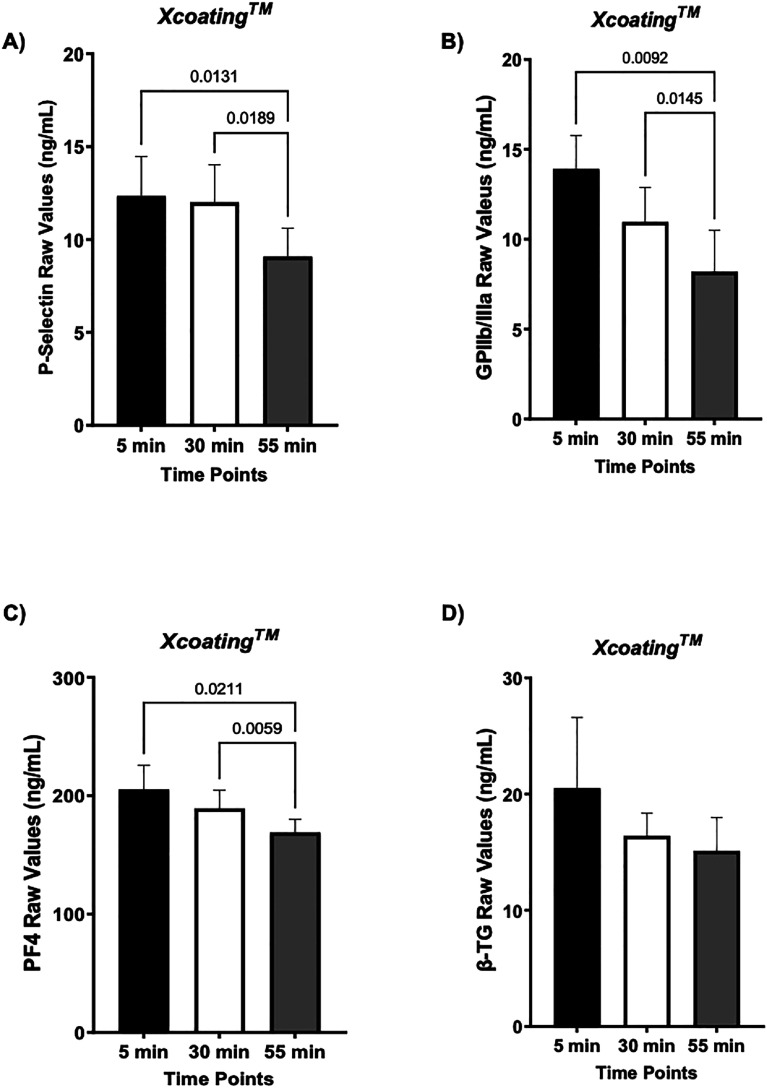
Raw values for platelet activation biomarkers using *Xcoating*^TM^ bypass. Bargraphs presenting raw values of platelet activation markers in collected bovine blood at 5-, 30-, and 55-minutes using *Xcoating*^TM^ bypass circuit without normalization over the platelet counts. (Mean ± SD, *P* < 0.05, five technical replicates). GPIIb/IIIa: Glycoprotein IIb/IIIa; PF4: Platelet Factor 4; β-TG: β-Thromboglobulin

The same blood collection methods and time points were used to evaluate the secretion and translocation levels of four platelet activation biomarkers using the *Xcoating*^TM^ bypass circuit. Our results show that normalized values (over platelet counts) for soluble P-selectin and PF4 translocation and release transiently but not significantly increased by 30 min, and returned to basal levels, with no statistically significant changes observed at 55 min (Figs. 4A and 4B), suggesting that luminal surface of *Xcoating*^TM^ tubes do not impact the level of soluble P-selectin and PF4 release in circulated blood. On the other hand, normalized values for GPIIb/IIIa showed a significant drop at 55 min in the *Xcoating*^TM^ circuit (Fig. 4C), with no significant changes observed for β-TG levels in the circulated blood at any time points (Fig. 4D). In addition, statistical analyses were performed using raw values for all four biomarkers secretion and platelet surface levels without normalization over the platelet counts in bypassed bovine blood at 5-, 30-, and 55-minutes in *Xcoating*^TM^ bypass circuit (Supplementary Fig. S2). A summarized version of all collected values for platelet activation markers in circulated bovine blood is also presented in Table 1.

**Table 1:** A collective list of values for four biomarkers of platelet activation using *Trillium*^®^ *Biosurface and Xcoating*^TM^ circuits. GPIIb/IIIa: Glycoprotein IIb/IIIa; PF4: Platelet Factor 4; β-TG: β-Thromboglobulin

Coating	Time point	P-selectin	β-TG	PF4	GPIIb/IIIa
*Trillium*^®^ *Biosurface* (Normalized)	5 min	0.311 ± 0.074	0.184 ± 0.025	2.322 ± 0.058	0.125 ± 0.01
	30 min	0.217 ± 0.061	0.183 ± 0.025	2.278 ± 0.079	0.111 ± 0.013
	55 min	0.221 ± 0.082	0.169 ± 0.024	2.113 ± 0.188	0.09 ± 0.012
*Xcoating*^TM^ (Normalized)	5 min	0.064 ± 0.011	0.107 ± 0.032	1.071 ± 0.107	0.073 ± 0.01
	30 min	0.081 ± 0.014	0.111 ± 0.013	1.277 ± 0.104	0.074 ± 0.013
	55 min	0.063 ± 0.011	0.105 ± 0.02	1.173 ± 0.07	0.057 ± 0.016

## Discussion

A cardiopulmonary bypass is a machine that allows for a surgical field suitable to perform open heart surgery, however, it is not devoid of complications [1]. CPB-induced SIRS affects up to 10% of patients and can lead to hypoperfusion, embolization, multiple organ failure, and death [13]. Several proinflammatory pathways work synergistically to cause tissue destruction, and abnormal bleeding [13]. Platelets are known to bind damaged blood vessels and cause coagulation [4]. Therefore, in cases of serious complications like CPB-induced SIRS, dysfunctional platelets are the likely reason for organ damage and excessive bleeding. Modern technology has introduced heparin-coated and PMEA-coated circuits to reduce platelet activation and control some of the complications associated with CPB. However, there is no established and well-controlled *in vitro* protocol that allows for the accurate assessment of the efficiency of commercially available bio-coated bypass circuits in blocking the CPB-mediated platelet activation in a surgical setting.

The main objective of this study was to establish a standardized method to study platelet activation *in vitro* for future comparative studies, and to further determine if there are any significant inter-variations across the circuit batches that would potentially impact the accuracy of not only the biomarker measurements across technical and biological replicates, but also the reliability of our statistical analyses. Moreover, the data collected in the current pilot study will allow for appropriate study sample size calculation that provides enough power for more reliable and reproducible comparative future studies across different types of biocoated CPB circuits.

In this study, we measured platelet counts and the secretion and translocation levels of known and well-established biomarkers of platelet activation including PF4, β-TG, GPIIb/IIIa, and soluble P-selectin at different time points of CBP. Together, these biomarkers provide a comprehensive profile of platelet activation, aggregation, adhesion, and subsequent coagulation cascade that is triggered by the contact of platelets with foreign surfaces such as the lumen of the CPB circuits.

PF4 is a cytokine released from alpha granules of platelets during activation and blood coagulation, and its increased level was found to be consistent with the incidence of cardiac complications during bypass surgeries [3]. In addition, PF4 was shown to be elevated in peripheral artery disease, acute ischemic stroke, essential thrombocytopenia, and other clinical conditions that were associated with platelet activation and is thus considered a relatively more sensitive marker of platelet activation during pathological events [14]. *Xcoating*^TM^ showed a decrease in PF4 total amount throughout the trial, however, when normalized with platelet count, there was an increase then decrease in PF4. Although there was an increase in PF4 normalized concentration, it remained unchanged at the end of the trial. *Trillium*^®^ *Biosurface* showed a constant PF4 concentration indicating that no further activation occurred to increase PF4.

In addition to PF4, β-TG is another cytokine released early following platelet adhesion and is commonly used as a reliable marker of platelet activation *in vitro* [14]. After the initial increase in β-TG concentration, *Trillium*^®^ *Biosurface* and *Xcoating*^TM^ did not have significant effects throughout the experiments. Another platelet marker GPIIb/IIIa belongs to the large family of integrin complexes and plays a critical role in platelet aggregation and increased binding to plasma fibrinogen and endothelial *von Willebrand* factor, therefore, facilitating thrombin generation and blocking hemorrhage [15]. Furthermore, GPIIb/IIIa surface translocation increases upon platelet activation making it a valuable biomarker in quantifying platelet activation and aggregation [16]. Both circuit coatings prevented further GPIIb/IIIa activation indicated by a decrease in concentration during the trials. Another platelet activation marker P-selectin is stored in alpha granules of platelets as well as in the Weibel-bodies of endothelial cells that cover the lumen of the blood vessels. Upon activation, P-selectin is translocated to the surface of endothelium and released into the blood circulation and plays an important role during the initial adhesion and rolling of platelets in the site of injury [17, 18]. In addition, activated platelets result in an increase in soluble P-selectin translocation and release allowing for quantification of platelet activation [19]. The inhibition of P-selectin release was displayed by a decrease in concentration over the course of the trial in *Trillium*^®^ *Biosurface*. When normalized over the platelet counts, soluble P-selectin levels did not show any significant changes throughout the experiment using *Xcoating*^TM^ circuits.

This was a longitudinal study that measured the platelet activation at different time points during a circuit run for two different types of CPB circuit coatings. The baseline measurement was arbitrarily set at 5 min, so there would be adequate time for the crystalloid to mix with the bovine blood within the circuit. Measurements done at 30 min and 55 min were compared to 5 min and shown to both fall within ±2 standard deviations of the mean at each time point for each type of coated circuit. ELISA assays were then utilized to measure platelet activation markers due to their high sensitivity and specificity. While these assays are not intended to be used in a clinical setting for patients undergoing CPB, they can provide insight into the activity of platelets within the CPB coating during cardiac surgery.

It is noteworthy, that although this study has shown minimum inter-variations within each type of coating, it cannot provide conclusive comparative data of any potential differences between the coating types. The present study was designed to establish a standardized protocol in a laboratory setting that would allow us to plan future experiments to compare different types of bio-coated circuits in the market and address a very critical question: which type of commercially available CPB circuit would provide better protection against platelet activation during human bypass surgeries? In addition, we were not able to have a positive control group for this study, as uncoated reservoirs or tubing are not available from the same vendor for clinical use. The use of custom-made uncoated CPB circuit would have added confounding variables to the study. Our established protocol remains accurate shown by the similarities in biomarker activation in two different circuit coatings and the precision, consistency, and duplicability of the ELISA results.

In this study, we were able to establish a laboratory protocol using bovine blood that minimizes the inter-variations within each type of circuit through different time points. The results presented in this report confirm that the method is precise, reliable, and reproducible given no inter-variation within each coating at different time points. The preliminary data collected in this study will be used for power analysis and sample size calculations to determine the proper sample size (numbers for technical and biological replicates) for future comparative studies.

## Data Availability

The raw original data used in this study is available by the authors upon request.

## Supplementary material

The Supplementary materials of this article are available at https://ject.edpsciences.org/10.1051/ject/2024003/olm.
